# Whole Genome Sequencing Reveals Clade‐Specific Genetic Variation in Blacklegged Ticks

**DOI:** 10.1002/ece3.70987

**Published:** 2025-02-11

**Authors:** Jacob Cassens, Adela S. Oliva Chávez, Danielle M. Tufts, Jianmin Zhong, Christopher Faulk, Jonathan D. Oliver

**Affiliations:** ^1^ Division of Environmental Health Sciences, School of Public Health University of Minnesota Minneapolis Minnesota USA; ^2^ Department of Entomology University of Wisconsin – Madison Madison Wisconsin USA; ^3^ Department of Infectious Diseases and Microbiology, School of Public Health University of Pittsburgh Pittsburgh Pennsylvania USA; ^4^ Department of Veterinary Tropical Diseases University of Pretoria Pretoria South Africa; ^5^ Department of Biological Sciences California State Polytechnic University Humboldt California USA; ^6^ Department of Animal Science, College of Food, Agricultural and Natural Resource Sciences University of Minnesota Minneapolis Minnesota USA

**Keywords:** adaptation, blacklegged ticks, genomics, *Ixodes scapularis*, mitogenome, selection

## Abstract

Ticks and tick‐borne pathogens represent the greatest vector‐borne disease threat in the United States. Blacklegged ticks are responsible for most human cases, yet the disease burden is unevenly distributed across the northern and southern United States. Understanding the genetic characteristics influencing phenotypic differences in tick vectors is critical to elucidating disparities in tick‐borne pathogen transmission dynamics. Applying evolutionary analyses to molecular variation in natural tick populations across ecological gradients will help identify signatures of local adaptation, which will improve control and mitigation strategies. In this study, we performed whole genome nanopore sequencing of individual (*n* = 1) blacklegged ticks across their geographical range (Minnesota, Pennsylvania, and Texas) to evaluate genetic divergence among populations. Our integrated analyses identified genetic variants associated with numerous biological processes and molecular functions that segregated across populations. Notably, northern populations displayed genetic variants in genes linked to xenobiotic detoxification, transmembrane transport, and sulfation that may underpin key phenotypes influencing tick dispersal, host associations, and vectorial capacity. Nanopore sequencing further allowed the recovery of complete mitochondrial and commensal endosymbiont genomes. Our study provides further evidence of genetic divergence in epidemiologically relevant gene families among blacklegged tick clades. This report emphasizes the need to elucidate the genetic basis driving divergence among conspecific blacklegged tick clades in the United States.

## Graphical abstract

In this study, we performed whole genome nanopore sequencing of individual blacklegged ticks across their geographical range to evaluate genetic divergence among populations. Our integrated analyses identified genetic variants associated with numerous biological processes and molecular functions that segregated across populations. Nanopore sequencing further allowed the recovery of complete mitochondrial and commensal endosymbiont genomes.

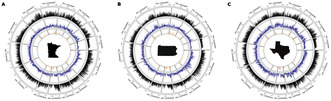

## 1 Introduction

Ticks pose a substantial threat to public health (Eisen and Eisen 2018). In the United States, eastern blacklegged ticks ( *Ixodes scapularis* ) and western blacklegged ticks ( *Ixodes pacificus* ) are the primary vectors of concern, with the ability to transmit seven human pathogens, including viruses, bacteria, and protozoa (Eisen and Eisen 2018). These vectors are responsible for most tick‐borne disease cases in the United States. *Ixodes scapularis*‐borne disease incidence is concentrated in the Upper Midwest and Northeast despite long‐established populations in the South (Eisen and Eisen 2018; Eisen and Eisen 2023), resulting from different questing and host preference behaviors between northern and southern populations (Arsnoe et al. 2015; Arsnoe et al. 2019; Ginsberg et al. 2021; Tietjen et al. 2020). Blacklegged ticks are obligate blood feeders, requiring a blood meal at each life stage for development and reproduction, and have been recorded infesting over 200 host species (Estrada‐Peña et al. 2023). Feeding for extended periods allows these ticks to disperse across environments, with their distribution and local abundance largely influenced by host mobility (Nadolny et al. 2015). Over the past two decades, blacklegged tick populations have expanded across North America through host‐mediated dispersal (Tsao et al. 2021). However, the successful colonization of new ecological niches following host‐mediated dispersal is likely shaped by the tick's genetic composition (De et al. 2021; De et al. 2023).

Blacklegged ticks belong to the Order Ixodida, Family Ixodidae, which consists of two groups: Prostriata and Metastriata (Guglielmone et al. 2010). The Prostriata group contains a single genus, *Ixodes*, which comprises 246 species (Mans et al. 2016; Klompen 2000). Historically, eastern blacklegged ticks in the United States were considered two distinct species: *Ixodes scapularis* in the South and *Ixodes dammini* in the North (Wesson et al. 1993). Subsequent mating experiments and phylogenetic studies demonstrated that these two species are conspecific (Oliver et al. 1993; Black and Piesman 1994). Following the synonymization of *I. dammini* and *I. scapularis* , researchers identified two distinct clades within *I. scapularis* : the All American clade in the North and the Southern clade in the South (Qiu et al. 2002; Rich et al. 1995; Norris et al. 1996). Although these clades were believed to be reproductively isolated, genetic analyses indicate admixture and gene flow at the periphery of their ranges (Van Zee et al. 2015; Xu et al. 2020; Frederick et al. 2023). Notably, Southern clade ticks exhibit higher genetic diversity, reflecting repeated demographic expansions and contractions at the northern front, potentially revealing their ancestral origins in North America (Xu et al. 2020; Sakamoto et al. 2014; Araya‐Anchetta et al. 2015).

Ticks from the All American clade exhibit distinct phenotypes in key traits compared to the Southern clade, which may influence tick‐borne disease dynamics across the United States. For instance, All American ticks have been observed to parasitize a broader range of hosts, including small, medium, and large mammals (e.g., mice, raccoons, deer) that serve as competent reservoirs for pathogens like *Borrelia*, while Southern clade ticks primarily infest non‐competent lizards (Eisen and Eisen 2023; Ginsberg et al. 2022). Although the drivers behind these host associations remain unclear, they may involve differences in questing behavior (Arsnoe et al. 2015; Arsnoe et al. 2019; Ginsberg et al. 2021; Ginsberg et al. 2014), environmental conditions (Ginsberg et al. 2017; Ginsberg et al. 2020), seasonal activity patterns (Ginsberg et al. 2021), and host specialization (Estrada‐Peña et al. 2023; McCoy et al. 2023). The genetic factors driving these patterns remain obscure. For example, genetic variation in genes (or their regulatory regions) underlying chemosensory receptors might alter how ticks detect host odors (Jia et al. 2020; Zhang 2024). Tick chemosensation involves the Haller's organ, the main sensory organ in ticks; however, little is known about the molecular mechanisms underlying tick chemoreception (Gebremedhin et al. 2023). Furthermore, molecular variation in genes associated with detoxifying host‐derived chemicals or metabolizing blood meal components (e.g., xenobiotic detoxification) may differ based on their evolutionary history (Jia et al. 2020; Schoville et al. 2024; De Araujo et al. 2024). This phenomenon is supported by a recent genome comparison of several tick species (Jia et al. 2020; Schoville et al. 2024). In particular, Southern clade ticks that feed on lizards, characterized by nucleated red blood cells and higher heme content, may face stronger selective pressures for enhanced heme metabolism (Whiten et al. 2018; Hajdusek et al. 2016; Donohue et al. 2009). The distinct ecological niches occupied by ticks impose unique selective pressures that drive local adaptation, often reflected in molecular variation (Jia et al. 2020; Schoville et al. 2024; Mccoy et al. 2002; McCoy et al. 1999). These molecular mechanisms collectively influence tick habitat distribution and vectorial capacity, shaping the evolving landscape of tick‐borne disease risk (De La Fuente et al. 2017).

Over the past decade, significant advances in blacklegged tick genomics have emerged, including high‐quality genome assemblies from ticks and the ISE6 tick cell line (De et al. 2023; Nuss et al. 2023; Miller et al. 2018). The blacklegged tick genome, spanning 2.2 Gb, consists of over 70% repetitive elements, which may contribute to their phenotypic plasticity and adaptability (De et al. 2023). The role of transposable elements in tick biology and adaptability still needs to be explored (Gilbert et al. 2021; Schrader and Schmitz 2019). Recent advances in sequencing technology, particularly nanopore sequencing, have transformed our ability to characterize tick genetic compositions. Nanopore sequencing, developed by Oxford Nanopore Technologies (ONT), utilizes an array of nanopores on a flow cell to sequence DNA molecules by measuring changes in ionic current as each molecule passes through a pore (Kipp et al. 2023). This platform uniquely allows the native sequencing of methylated cytosines alongside unmodified bases, providing a powerful tool for genomic and epigenomic analysis. Its capacity to produce long reads makes nanopore sequencing particularly suitable for the large and complex genomes of *Ixodes* ticks.

In this study, we employed nanopore sequencing to investigate the genetic and mitogenomic variation in *I. scapularis* and *I. pacificus* , the primary vectors of tick‐borne diseases in North America. By sequencing individuals from geographically distinct populations, we assessed variability within and between species, offering insights into population structure and evolutionary dynamics. We sequenced individual (*n* = 1) blacklegged ticks from Minnesota, Pennsylvania, and Texas to evaluate genetic variation in geographically isolated populations that represent the two distinct clades (All American and Southern) throughout the United States. This approach generated high‐resolution data on genetic diversity, potentially linked to phenotypic differences. We also assembled and analyzed complete mitochondrial and endosymbiont genomes to explore phylogenetic relationships. These integrated analyses aimed to uncover genetic factors contributing to phenotypic variation relevant to disease transmission, advancing our understanding of how molecular variation influences tick biology.

## 2 Methods

### 2.1 Sample Collection

Questing ticks were collected by dragging a 1‐m^2^ cloth along natural vegetation, stopping every *~*10 m to collect attached ticks, from four locations throughout the United States: Minnesota (Anoka County, MN, USA), Pennsylvania (Allegheny County, PA, USA), Texas (Polk County, TX, USA), and California (Riverside County, CA, USA). Collected ticks were preserved in 70% EtOH or RNAlater and sent to the University of Minnesota for processing. Ticks were morphologically identified using keys from Keirans and Clifford (1978), and Cooley and Kohls (1945). Ticks were rinsed with molecular grade water and transferred to Zymo DNA/RNA shield until DNA extraction.

### 2.2 DNA Extraction and Sequencing

DNA was extracted using a Qiagen MagAttract kit (Cat. 67563; Aarhus, Denmark) according to the manufacturer's instructions, with a final elution step extended to 1 h at 37°C. DNA was sheared by 20 passes through a standard 30‐gauge insulin syringe. Library prep was performed with an SQK‐LSK‐114 native ligation sequencing kit (ONT, Oxford, UK). Sequencing was performed on a P2 Solo instrument and basecalled using Nvidia 4090 GPUs. Sequencing was performed over 3 days with nuclease flush and library reload every 24 h. Individual adult females were used for sequencing of *I. scapularis* and *I. pacificus* (*n* = 1) from each location.

Raw data from all runs were basecalled together using Dorado v0.8.1 with the “super accuracy” model dna_r10.4.1_e8.2_400bps_supv4.2.0. Read quality was assessed using Nanoq v0.10.0 (Steinig and Coin 2022).

### 2.3 Variant Analysis

#### 2.3.1 Variant Calling

Super‐high accuracy basecalled reads from the three female adult *I. scapularis* (MN, PA, TX) specimens were individually mapped to the *I. scapularis* reference genome, PalLabHiFi (GCF_016920785.2) (De et al. 2023), using Minimap2 v2.28 (Li 2018). The resulting mapped bam file was sorted and indexed using Samtools v1.20 (Li et al. 2009) and served as input to call variants with Clair3 v1.0.10 (Zheng et al. 2022). Variants with quality scores of < 10 and allele frequencies of < 20% were masked using BCFtools v1.20 (Danecek et al. 2021). Filtering variants with a minor allele frequency of 20% and a quality score of 10 reflects a balance between excluding likely sequence errors and retaining biologically meaningful variation. Filtered variant call files generated alternative whole genome assemblies for each tick by replacing variant sites in the reference assembly using BCFtools. Variants were not called for the *I. pacificus* individual, as the reference assembly was scaffolded using the *I. scapularis* reference genome, limiting our confidence in the variants called for this sister species.

#### 2.3.2 Variant Statistics

**FIGURE 1:**
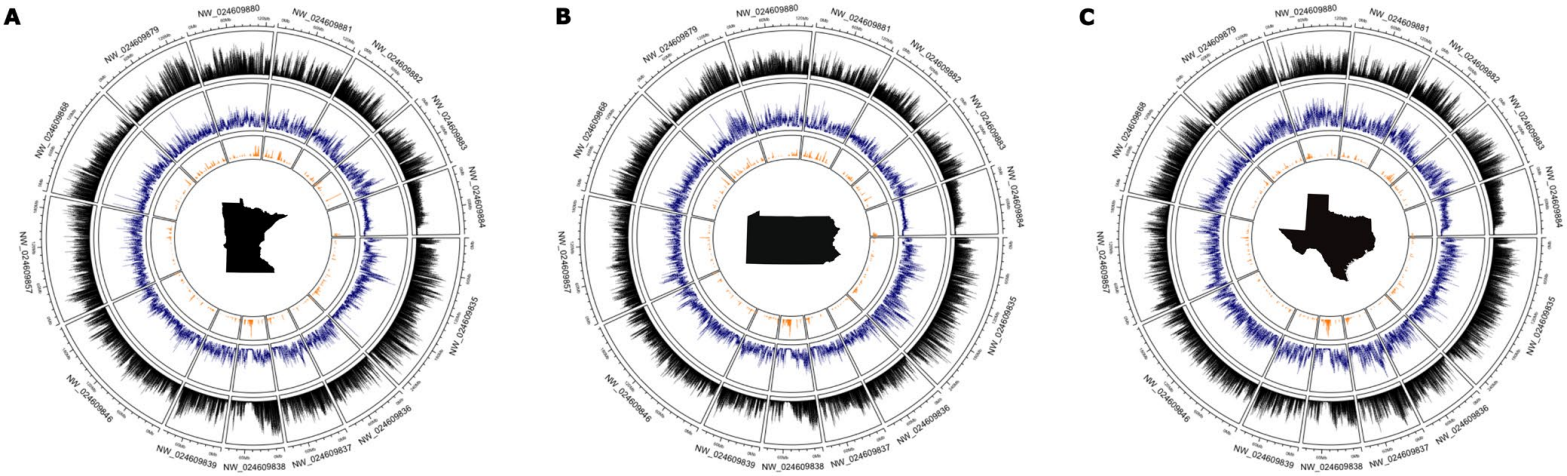
Circular visualization of genome‐wide SNP density (black), nucleotide diversity (blue), and runs of homozygosity (orange) for Minnesota (A), Pennsylvania (B), and Texas (C) *Ixodes scapularis* individuals by the 14 longest scaffolds in the genome.

Summary statistics were generated for each variant call file using VariantQC v1.05 (Yan et al. 2019) and Nanoq (Steinig and Coin 2022). Genome‐wide heterozygosity and SNP density were calculated in 100 kb windows as a proxy for genome‐wide diversity using VCFtools v0.1.16 (Danecek et al. 2011). Runs of homozygosity (ROH) were analyzed with Plink v1.90 (Purcell et al. 2007) specifying a sliding window of 100 kb, a threshold of 0.05 for overlapping homozygous windows, a minimum of 100 homozygous SNPs per window, a minimum SNP density of 50 kb per SNP, a minimum of 25 homozygous SNPs per ROH, a maximum of one heterozygous position per window, a maximum of 1000 kb gap between SNPs, and a maximum of 100 heterozygous sites in each final ROH (Dehasque et al. 2024). Genome‐wide heterozygosity, SNP density, and runs of homozygosity were visualized using circlize v0.4.15 (Gu et al. 2014) in R v4.2 (R Core Team 2022) (Figure 1).

#### 2.3.3 Variant Annotation

Filtered variant files were annotated using snpEff v5.2 (Cingolani, Platts, et al. 2012). SnpEff requires an annotation database to perform variant effect prediction. We built a custom database by specifying the reference genome (GCF_016920785.2), including the FASTA and GTF annotation file from NCBI. SnpEff annotates variants and predicts the coding effects of genetic variants by creating a data structure that algorithmically identifies intersecting genomic regions to calculate variant effects (Cingolani, Platts, et al. 2012). Annotated variant call files were sifted using snpSift (Cingolani, Patel, et al. 2012) to partition variant annotations, including the impact and effect of each variant, by the 14 longest scaffolds in the PalLabHiFi assembly. Annotation summary statistics for the whole genome and scaffolds were generated by snpSift.

#### 2.3.4 Gene Ontology

Gene ontology (GO) analysis was performed using the Database for Annotation, Visualization, and Integrated Discovery v2023q4 (DAVID) (Sherman et al. 2022; Huang et al. 2009). GO discovery was performed on two curated datasets. The first dataset was established to identify regions putatively identical by descent among the three female adult *I. scapularis* individuals (MN, PA, TX). To do so, runs of homozygosity shared across all three individuals were identified using dplyr v1.1.4 (Wickham et al. 2023) in R. Genes falling within these shared runs of homozygosity were intersected with gene annotation files using BedTools v2.31.1 (Quinlan and Hall 2010) to produce a dataset that contained locations of genes, with their respective gene IDs, shared across the individuals. The second dataset included all genes annotated as having a high impact through snpEff. The gene IDs from the two datasets were then independently used as input into DAVID and extracted as tables. GO terms shared among all individuals, some individuals, or unique to an individual were investigated. GO terms were visualized using ggVennDiagram v1.5.2 (Gao et al. 2021) and ggplot2 v3.5.1 (Wickham 2016).

### 2.4 Mitogenome Assembly, Annotation, and Phylogenetics

The mitogenomes of the three *I. scapularis* (MN, PA, TX) and *I. pacificus* (CA) individuals were extracted from their respective assemblies and characterized with MitoHiFi v3.2.2 (Uliano‐Silva et al. 2023). MitoHiFi identifies mitochondrial contigs from whole genome sequencing datasets, assembles the genome, and annotates it for protein and RNA genes through external tools (e.g., minimap2, Hifiasm, MitoFinder, etc.) (Uliano‐Silva et al. 2023). MitoHiFi was explicitly designed to handle long‐read sequencing data. To ensure proper assembly and annotation, the consensus FASTA generated from MitoHiFi was polished using Medaka v2.0.1 (https://github.com/nanoporetech/medaka) and annotated using Mitos2 v2.1.9 (Bernt et al. 2013) with specifications for metazoan RefSeq and invertebrate genetic code. Manual annotation correction was performed to correct frameshift mutations resulting in premature stop codons within highly conserved protein‐coding genes to ensure consistency with conserved mitochondrial gene structures. All frameshift mutations were found in homopolymer regions (i.e., stretches of consecutive adenine and thymine), a known limitation of nanopore sequencing. Manual correction was also performed to confirm tRNA annotations or added if any annotations were absent. Any tRNA annotation additions were performed using Geneious Prime software v2024.0.7 (https://www.geneious.com) by aligning the gene in question from reference mitogenomes with our polished mitogenome assembly using minimap2. Polished and assembled mitogenomes were visualized using OGDRAW v1.3.1 (Greiner et al. 2019).

After the mitogenomes were annotated, each mitogenome, cytochrome c oxidase subunit I (COI), and 16S gene sequences were used in phylogenetic analyses to determine their relatedness to congeneric *Ixodes* spp. To build the phylogenies, COI, 16S, and mitogenome sequences were downloaded from GenBank for each available North American *Ixodes* spp. The genus *Ixodes* (Acari: Ixodidae) is monophyletic and shares a most recent common ancestor with Metastriata, which resides within the same family, Ixodidae. As such, the metastriate species *Amblyomma americanum* was chosen as the outgroup and included in each analysis to root the phylogeny. Sequences were aligned using CLUSTALO v.1.2.3 (Thompson et al. 1994), and alignments were used in maximum likelihood analyses in RAxML v8.0.0 (Stamatakis 2014) with the GTRGAMMAI model of evolution and 1000 bootstrap replicates. The final tree was visualized in FigTree v1.4.4 (http://tree.bio.ed.ac.uk/software/figtree/).

### 2.5 Endosymbiont Genome Assembly, Annotation, and Phylogenetics

The genus *Rickettsia* has an intimate relationship with *Ixodes* spp., specifically *R. buchneri* with *I. scapularis* and *R. monacensis* strain Humboldt with *I. pacificus* (Kurtti et al. 2015; Hunter et al. 2015). Total reads from the three *I. scapularis* (MN, PA, TX) and *I. pacificus* (CA) tick whole genome assembly were individually mapped to the *R. buchneri* PalLab isolate genome (GCF_000696365.2) and *R. monacensis* isolate IrR/Munich reference genome (GCF_000499665.2), respectively, using minimap2. Mapped reads were used to assemble the *Rickettsia* spp. genomes with Flye v2.9.5 (Kolmogorov et al. 2019), followed by two rounds of polishing with Medaka. Whole genome assemblies for *R. buchneri* and *R. monacensis* were extracted from the raw sequencing files. Comparison of the draft genomes with other rickettsial species was conducted by extracting and concatenating 11 protein‐coding genes for phylogenetic analysis described in Kurtti et al. (2015). Sequences were aligned using CLUSTALO v.1.2.3 (Thompson et al. 1994), and alignments were used in maximum likelihood analyses in RAxML v8.0.0 (Stamatakis 2014) with the GTRGAMMAI model of evolution and 1000 bootstrap replicates. The final tree was visualized in FigTree v1.4.4 (http://tree.bio.ed.ac.uk/software/figtree/).

## 3 Results

### 3.1 Sequencing

Whole genome sequencing was performed to investigate genetic variation across three locations (MN, PA, and TX) within the geographic range of blacklegged ticks in the United States. After quality filtering, the sequencing runs generated 55 million reads and 94 Gb of sequence, yielding between 18 and 29 Gb for individual ticks (Table 1). The read N50 for *I. scapularis* ranged from 2.7 to 5.8 kb, with maximum read lengths reaching 137.5–420 kb (Table 1).

**TABLE 1:** Nanopore sequencing read summary statistics for three *Ixodes scapularis* individuals and one *Ixodes pacificus* individual.

Organism	Location	Read count (Mb)	Base count (Gb)	Coverage	N50	L50	Q20 (%)	Q30 (%)	Avg. quality	GC (%)
*I. scapularis*	Minnesota	17.3	29.6	13×	2784	42,534	78.6	63.9	15.0	45.1
*I. scapularis*	Pennsylvania	10.1	20.5	9×	4718	29,491	80.9	67.0	15.4	45.5
*I. scapularis*	Texas	9.4	25.9	12×	5840	31,183	80.0	65.2	15.5	45.9
*I. pacificus*	California	18.2	18.3	8×	1316	30,123	77.1	61.7	14.6	44.7

### 3.2 Variant Analysis

#### 3.2.1 Detecting Variants in Genome Sequences

After mapping the reads to the PalLabHifi reference assembly, we identified 16, 13, and 19 million sequence variants in the Minnesota, Pennsylvania, and Texas individuals, respectively (Table 2). Most variants were single nucleotide polymorphisms (SNPs), with a smaller fraction representing small insertions and deletions (indels; Table 2). Heterozygosity for SNPs and indels was highest in Texas, intermediate in Pennsylvania, and lowest in Minnesota (Table 2). Detailed information regarding variant statistics for the whole genome and each of the 14 longest scaffolds is provided in Tables S1 and S2. Sliding window nucleotide diversity estimates corroborated these findings, showing nearly identical values for Minnesota and Pennsylvania (0.0025 and 0.0026) and significantly higher diversity in Texas (0.0044; Table S3). Cumulative runs of homozygosity emphasized this trend, revealing similar patterns for Minnesota and Pennsylvania, which were markedly different from Texas (Table S3). The average SNP density was highest in Texas, intermediate for Minnesota, and lowest for Pennsylvania, likely reflecting the reduced sequence data obtained from Pennsylvania (Table S3). The distribution of SNPs, nucleotide diversity, and runs of homozygosity within 100 kb windows across the 14 longest scaffolds in the *I. scapularis* genome is presented in Figure 1. Overall, the longest scaffold (NW024609835) contained the highest number of variants, while the shortest scaffold (NW024609884) had the fewest across all organisms (Table S2).

**TABLE 2:** Variant statistics for the three *Ixodes scapularis* individuals called using Clair3. Variants were filtered for a minor allele frequency of 20% and quality score of 10.

Location	# of SNPs	# of indels	# of variants	Variant density per kb	Het/Hom for SNPs	Het/Hom for Indels	Ti/Tv	# of Called genotypes
Minnesota	15,487,600	1,045,673	16,533,273	0.53	0.49	0.36	1.52	16,442,467
Pennsylvania	12,285,692	715,169	13,000,861	0.41	0.60	0.61	1.56	12,929,778
Texas	17,854,460	1,242,919	19,097,379	0.61	0.86	0.73	1.59	18,977,349

#### 3.2.2 Predicting Variant Effects on Gene Function

The effects of sequence variants on gene function were predicted using the SnpEff program, which categorizes variants into four groups: high, moderate, low, and modifier. Most variants were predicted to modify gene function, primarily consisting of intergenic, intronic, and upstream gene variants (Table 3). Low‐impact variants were predominantly synonymous mutations, while moderate‐impact variants were almost entirely missense mutations (Table 3). Frameshift mutations were the most common high‐impact variant across all individuals, followed by stop codon gains and splice donor variants (Table 3). High‐impact variants were less common than the other three groups, and their distribution among northern ticks showed heterogeneity across the 14 longest scaffolds. In Texas, high‐impact variants were homogenously distributed, primarily from intermediate‐length scaffolds (Table S4). To identify high‐impact sequence variants in genic regions, we extracted these variants from each individual's variant call file. We intersected them with publicly available gene annotation files from the PalLabHifi reference assembly, which contains 38,656 annotated genes including 26,659 protein‐coding genes (De et al. 2023). Our analysis focused on the 14 longest scaffolds, which are believed to represent the karyotype of blacklegged ticks (De et al. 2023).

**TABLE 3:** Classification of variants by their effects for the three *Ixodes scapularis* individuals. Variant effects were predicted using SnpEff.

Impact of SNP effect	SNP effect	Number		
		Minnesota	Pennsylvania	Texas
High	Frameshift mutation	619	445	873
	Stop codon gained	403	326	536
	Splice donor variant	254	193	279
	Stop codon lost	199	154	218
	Splice acceptor variant	192	155	204
	Start codon lost	132	99	141
	Gene fusion	4	1	4
	Exon loss variant	—	—	1
	Sum	1803	1373	2256
Moderate	Missense mutation	47,274	35,977	49,444
	Inframe indel mutation	736	574	879
	Sum	48,010	36,551	50,323
Low	Synonymous mutation	89,709	69,571	89,361
	Splice region variant	18,910	15,120	20,158
	5' UTR premature start codon gained	3228	2367	3256
	Missense mutation	306	256	302
	Stop codon retained	77	68	81
	Initiator codon variant	18	17	15
	Splice acceptor variant	11	14	11
	Splice donor variant	12	12	10
	Stop codon gained	11	8	8
	Inframe indel mutation	2	3	6
	Frameshift mutation	2	2	4
	Stop codon lost	7	—	3
	Gene fusion	1	—	1
	Start codon lost	—	4	—
	Sum	112,294	87,442	113,216
Modifier	Intergenic variant	8,153,255	6,431,461	9,389,028
Modifier	Intronic variant	5,806,150	4,554,040	6,754,669
	Upstream gene variant	1,081,097	843,663	1,244,723
	Downstream gene variant	853,712	671,001	986,854
	Intragenic variant	251,095	203,088	296,904
	3' UTR variant	109,725	86,586	114,135
	5' UTR premature start codon gain	25,639	18,015	25,342
	Synonymous mutation	19,002	13,759	18,291
	Missense variant	10,579	8047	11,154
	Splice region	3637	2805	3841
	Non coding transcript exon variant	3265	2397	3491
	Frameshift mutation	213	165	306
	Inframe indel mutation	196	154	259
	Stop codon gained	112	110	137
	Splice donor	83	69	88
	Splice acceptor	66	47	67
	Stop codon lost	57	43	61
	Start codon lost	47	33	48
	Stop codon retained	38	28	42
	Initiator codon variant	4	4	4
	Gene fusion	1	1	4
	Start codon retained	—	2	2
	Sum	16,317,973	12,835,518	18,849,450

#### 3.2.3 Predicting Functions of Gene Products

**FIGURE 2:**
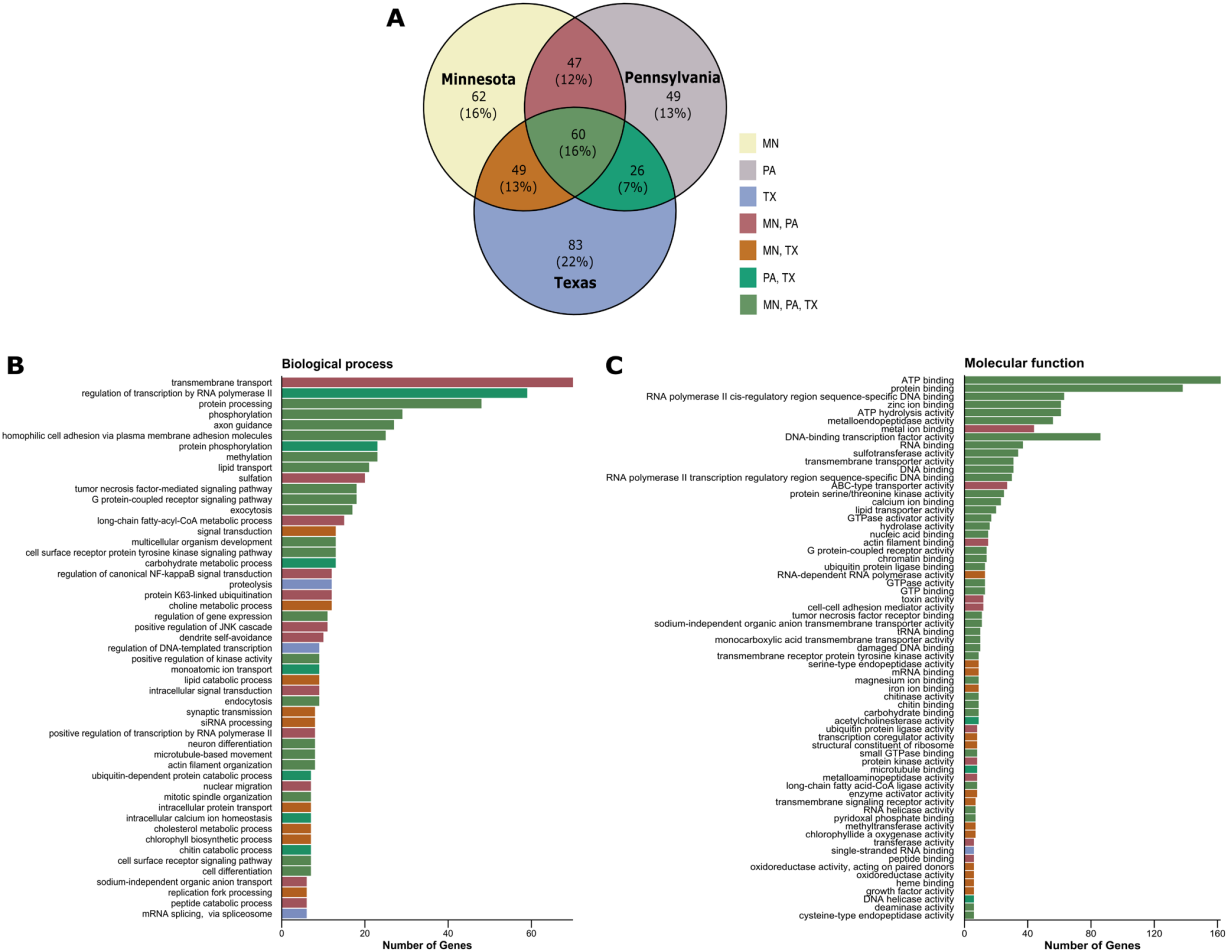
Gene ontology comparison for variants predicted to have high impact on gene function. (A) The comparison of high‐impact variant gene ontology terms across the three *Ixodes scapularis* individuals. Gene ontology terms were predicted using DAVID. Those gene ontology terms unique to an individual, shared among two individuals, or shared among all three individuals are differentiated by the colors described in the legend. (B) The biological process gene ontology terms and (C) the molecular function gene ontology terms for high‐impact variants. The colors of the bars in (B) and (C) correspond to the legend in (A).

The gene identification values of genes containing high‐impact variants were used to predict gene function with DAVID's GO tool. Comparative analysis across individuals revealed shared and unique high‐impact sequence variant GO terms (Figure 2). Texas blacklegged ticks exhibited the most unique terms, followed by Minnesota and Pennsylvania (Figure 2A). Most unique GO terms contained fewer than five genes and are not shown here; additional data can be found in Table S5. Sixteen percent of GO terms were shared among all three individuals (Figure 2A) and are predicted to be involved in various molecular functions and biological processes, notably cell signaling and receptor activity (e.g., G‐protein‐coupled receptor, signal transducers), transcriptional regulation (e.g., methylation, transcription factor activity, RNA pol II), binding activities (e.g., ATP and protein binding), and hydrolase and kinase activity (e.g., metalloendopeptidase) (Figure 2B,C). We were particularly interested in GO terms shared among northern blacklegged ticks, which may indicate genes with different evolutionary histories compared to southern ticks. Twelve percent of GO terms were shared between Minnesota and Pennsylvania only (Figure 2A). Shared genetic variants related to biological processes among northern ticks include cellular transport, signal transduction, and metabolic regulation (Figure 2B). Molecular function genetic variants are predicted to affect protein function, enzymatic activity, and cellular interaction (Figure 2C). Notably, northern ticks exclusively shared high‐impact variants in genes involved in xenobiotic detoxification, such as sulfation, ABC‐transporter, and toxin activity (Figure 2). Additionally, GO terms shared by Minnesota and Texas only represented the second highest proportion of GO terms (13%), containing variants in genes associated with heme and iron ion binding (Figure 2). GO terms involving less than five genes were separated for brevity and are available in Table S5 and Figure S1.

**FIGURE 3:**
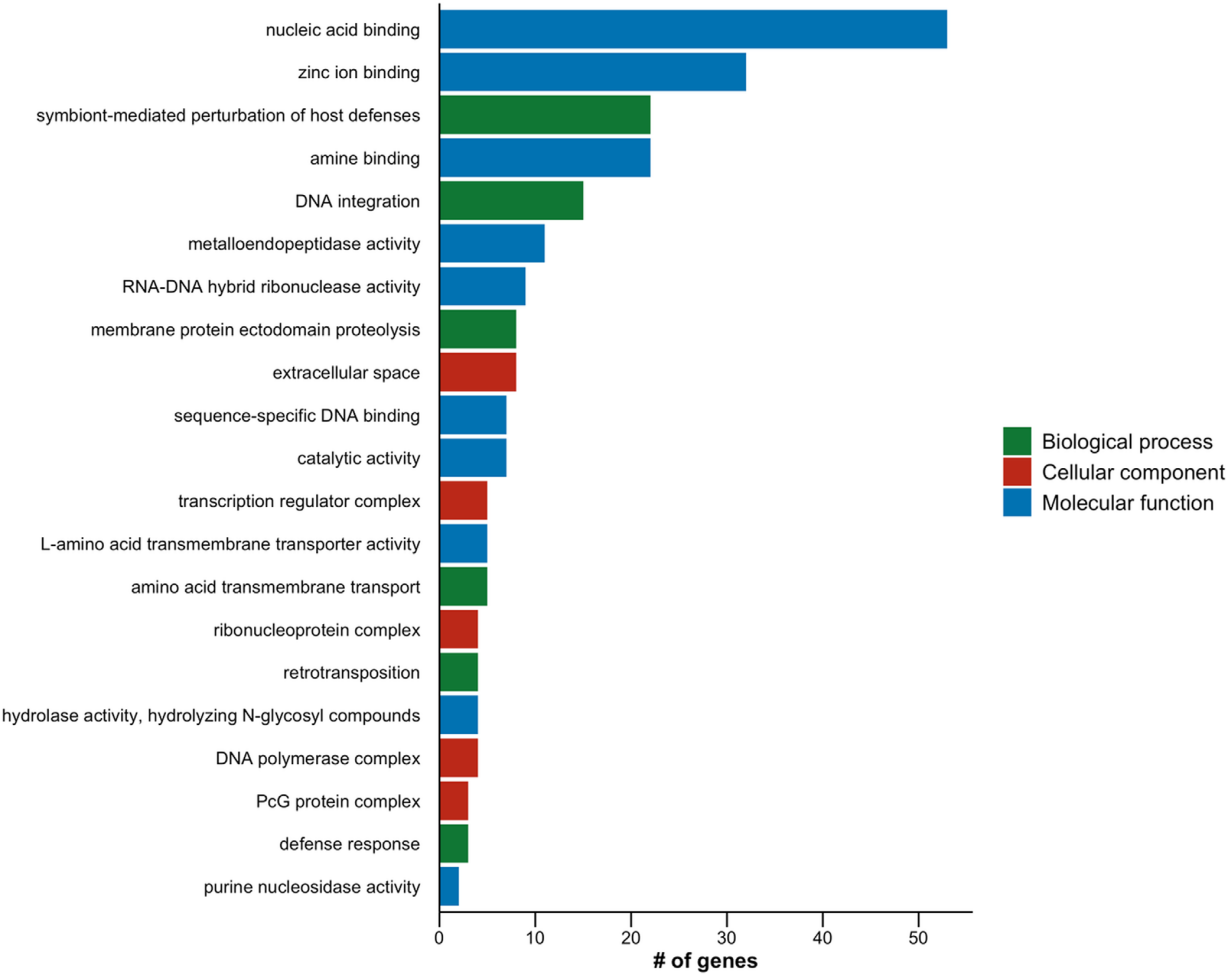
Gene ontology terms for genes found in shared runs of homozygosity across the three *Ixodes scapularis* individuals. Shared runs of homozygosity were used to infer genomic regions putatively identical by descent.

We also investigated genic regions that contained no variants and were identical by descent across the three individuals. To do this, we identified runs of homozygosity shared among the three individuals, intersected these regions with gene annotation files from the PalLabHifi reference assembly, and predicted gene function using the pipeline described above. Genomic regions identical by descent between northern and southern ticks—presumably under purifying selective pressure—include genes involved in genetic material processing, protein activity, transport, binding, and cellular structures (Figure 3).

## 4 Mitochondrial Genome

**FIGURE 4:**
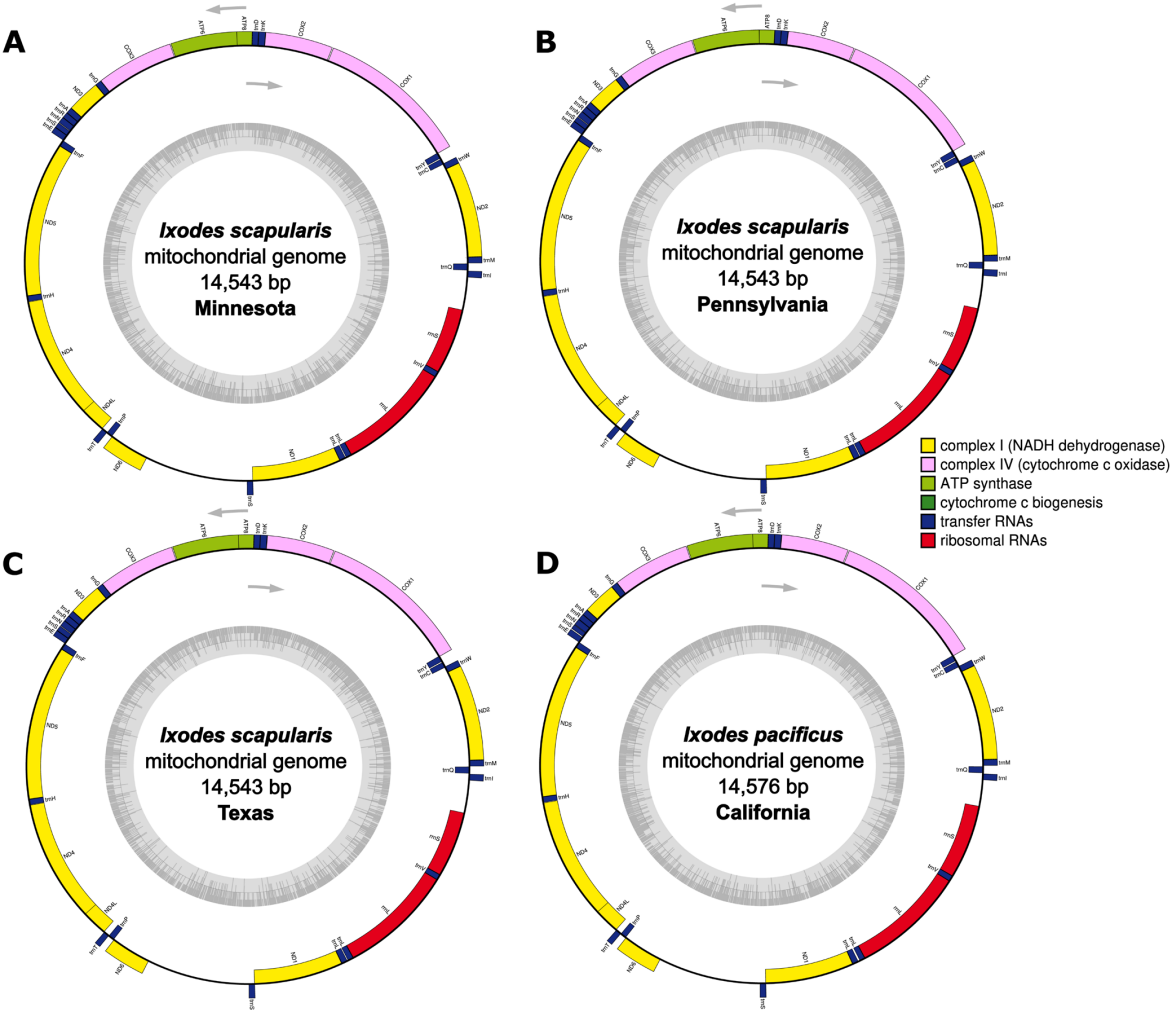
Nanopore mitochondrial genome assemblies and annotations of the three *Ixodes scapularis* from Minnesota (A), Pennsylvania (B), and Texas (C), and the *Ixodes pacificus* from California (D).

**FIGURE 5:**
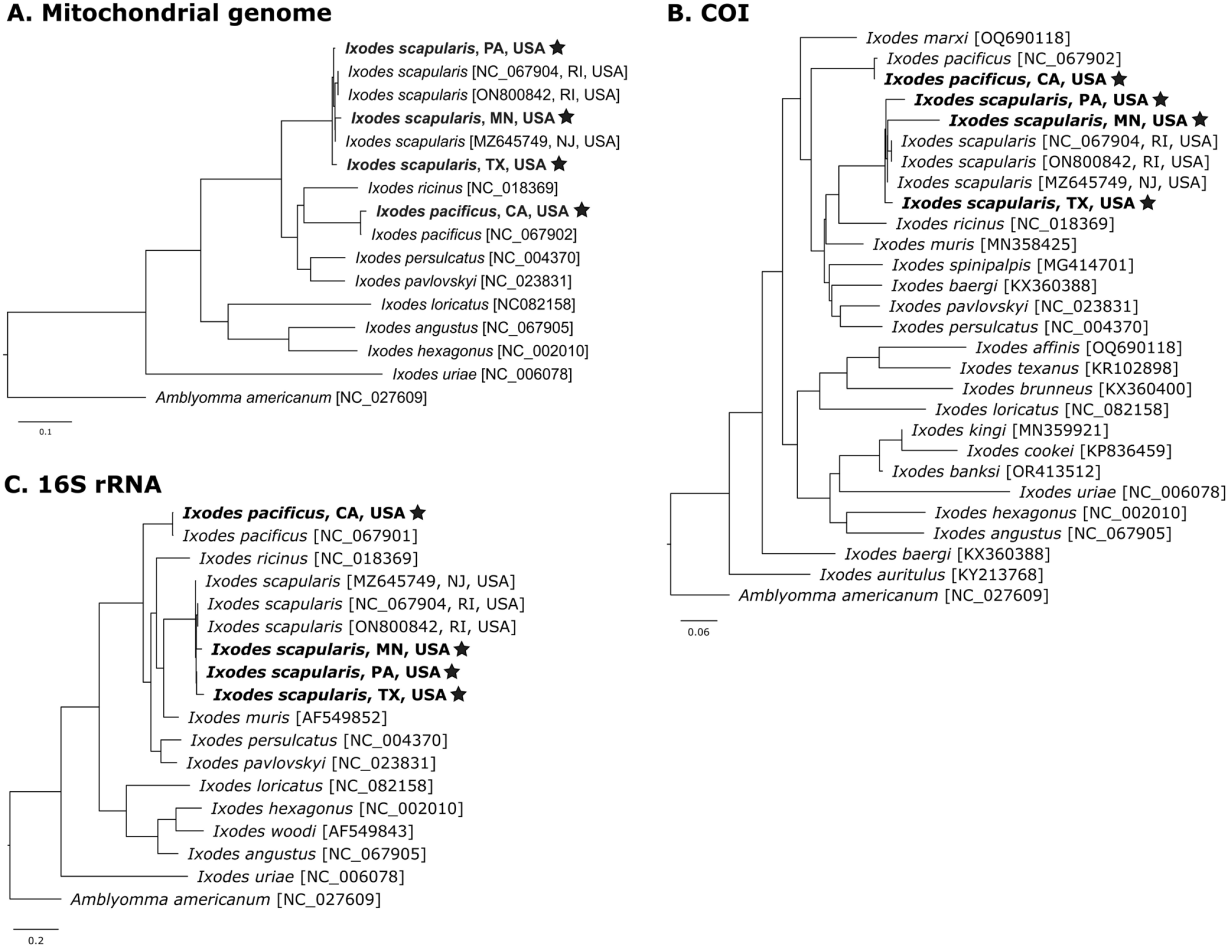
Phylogenetic relationships of *Ixodes scapularis* and *Ixodes pacificus* were derived through bootstraped maximum likelihood comparison of the assembled mitogenomes to existing hard tick references (Ixodida: Ixodidae). Phylogenies were created using the entire mitochondrial assembly (A), the small ribosomal RNA gene (B), and the cytochrome c oxidase gene (C), starred terms represent the organisms sequenced in this study.

The mitochondrial genome was extracted from the assembly and characterized using a combination of long‐read mitochondrial contig identification and annotation tools. The mitogenomes of *I. scapularis* differed by a few base pairs due to homopolymer errors in the D‐loop (Figure 4). The *I. scapularis* mitogenomes from Minnesota and Pennsylvania exhibited > 99.3% similarity to *I. scapularis* reference mitogenomes from Rhode Island (NC_067904 and ON800842) and New Jersey, USA (MZ645749). The *I. scapularis* mitogenome from Texas was 97% similar to these reference genomes and the *I. scapularis* individuals from Minnesota and Pennsylvania. Depth of coverage ranged from 5070× (PA), 4380× (TX), and 1250× (MN) for *I. scapularis* and 2310× (CA) for *I. pacificus* . The mitogenomes contained 22 tRNAs, 13 protein‐coding genes, and 2 rRNAs, conforming to the ancestral arthropod mitogenome organization. Our phylogenetic analysis of the entire mitogenome, COI, and 16S rRNA confirmed the monophyly of *I. ricinus* group ticks, with all *I. scapularis* ticks forming a distinct grouping (Figure 5). The closely related *I. pacificus* also formed a distinct clade (Figure 5). All final mitochondrial genomes assembled in this study have been deposited in GenBank (accession numbers: PQ557589‐PQ557592).

### 4.1 Endosymbiont Genome

**FIGURE 6:**
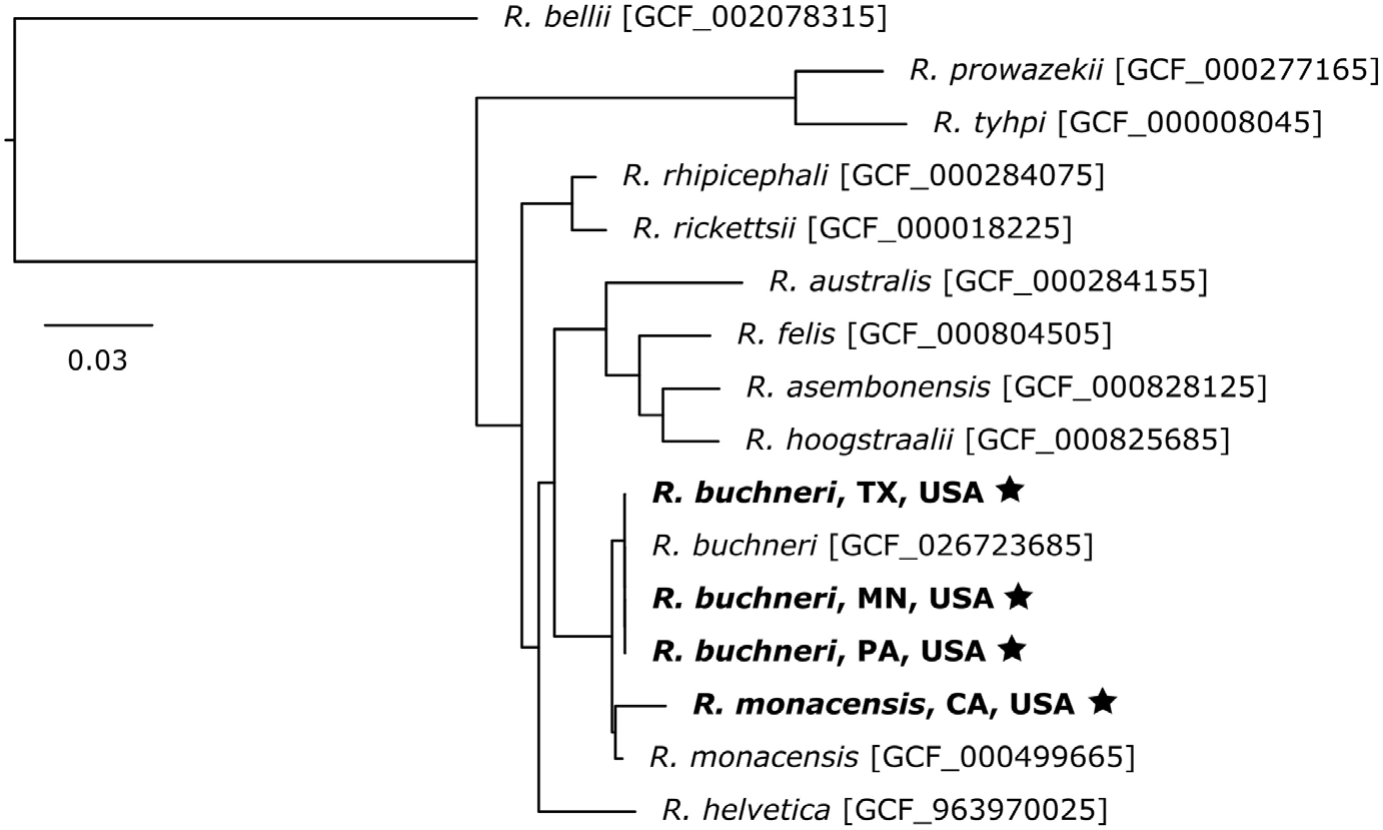
Phylogenetic relationships of Rickettsia buchneri and Rickettsia monacensis present in the sequenced ticks were derived through bootstrapped maximum likelihood comparison of 11 concatenated proteins to existing rickettsiae references. Phylogenies were created using concatenated protein sequences in the order HtpG, InfB, RpoA, RpoB, PolA, ThrS, GroEl, RecAm DnaE, and Pnp. Trees were rooted by forcing *Rickettsia bellii* as the outgroup.

Commensal bacteria form intimate relationships with hard ticks, potentially providing nutritional supplementation in their strict blood‐feeding diet (Duron 2024). We were able to recover whole genome sequences for the primary commensal endosymbionts of *I. scapularis* and *I. pacificus* . Each *I. scapularis* individual and the *I. pacificus* individual yielded a single full‐length contig spanning the *R. buchneri* and *R. monacensis* chromosome, respectively. Depth of coverage ranged from 24.5× (MN), 22.9× (PA), and 17.2 (TX) for *R. buchneri* assemblies and 22.3× (CA) for *R. monacensis* . Phylogenetic analysis revealed that *R. buchneri* and *R. monacensis* recovered from naturally infected *Ixodes* ticks were closely related and group into a clade separate from other rickettsiae (Figure 6). The *R. buchneri* from *I. scapularis* individuals were > 99% similar to the PalLab isolate (GCF_026723685) and *R. monacensis* from the *I. pacificus* individual was 96% similar to the IrR/Munich isolate (GCF_000499665).

## 5 Discussion

This study examined the genetic composition of blacklegged ticks across their geographic range in the United States, with a focus on uncovering genetic differences between the All American and Southern clades that may influence their capacity to transmit pathogens. We focused our sequencing efforts on blacklegged tick populations from the All American (Minnesota and Pennsylvania) and Southern clades (Texas) to represent geographically segregated populations displaying divergent phenotypes. Previous research identified phenotypic differences between these clades, including variations in questing behavior, phenology, host preferences, and local abundances, which could explain regional disparities in tick‐borne disease incidence (Arsnoe et al. 2015; Arsnoe et al. 2019; Ginsberg et al. 2021; Tietjen et al. 2020; Estrada‐Peña et al. 2023; Nadolny et al. 2015; De et al. 2021; De et al. 2023). Specifically, these populations were chosen as Midwestern and Northeastern populations are thought to have originated from Southeastern populations, yet these populations show geographical variation both within and between clades in key life‐history characteristics (e.g., seasonal activity, questing behavior, host predilection) and contributions to tick‐borne disease (Eisen and Eisen 2018; Arsnoe et al. 2015; Arsnoe et al. 2019; Ginsberg et al. 2021; Tietjen et al. 2020; Estrada‐Peña et al. 2023; Nadolny et al. 2015; De et al. 2021; De et al. 2023). However, the genetic basis underlying these phenotypic traits remains largely unexplored. Our study addressed this gap by investigating genetic variation that may influence the vectorial capacity of these two clades, potentially elucidating their contribution to differences in disease transmission patterns across the United States. This highlights the necessity in adopting genetic approaches to investigate the underlying basis of tick vectorial capacity and phenotypic divergence.

Southern clade ticks from Texas displayed higher nucleotide diversity, heterozygosity to homozygosity ratios, and SNP density, whereas All American clade ticks exhibited longer and more frequent runs of homozygosity (Figure 1 and Table S3). These findings align with population genetic analyses showing that Southern clade ticks possess relatively high genetic diversity (Van Zee et al. 2013). Runs of homozygosity (ROH) are continuous lengths of DNA with homozygous sites across all loci that arise from identical haplotypes inherited from both parents (Ceballos et al. 2018). They reflect both individual and population history, serving as indicators of inbreeding. Shared ROH across individuals can reveal regions that are identical by descent, meaning they were inherited from a common ancestor without recombination. Shared ROH across the three eastern blacklegged tick individuals included genes involved in genetic material processing, protein activity, transport, binding, and cellular structures–core functions essential for tick fitness (Figure 3). Interestingly, shared ROH included genes belonging to the polycomb group (PcG) protein complex and transcription regulator complex, illustrating that gene regulation is multifaceted in tick genomes. PcG proteins regulate chromatin structure and gene expression, which could play a role in modulating transcriptional responses to environmental pressures or developmental needs (De et al. 2023). These conserved genomic regions, likely under strong purifying selection, highlight fundamental molecular functions and biological processes. The longer stretches and frequencies of ROH in All American clade ticks may result from recurrent reductions in effective population sizes (e.g., population bottlenecks or founder effects) experienced by these expanding populations, which limit genetic diversity, suggesting that Southern clade ticks could represent the ancestral origin of blacklegged ticks in the United States (Xu et al. 2020; Frederick et al. 2023). Moreover, contrasting ecological conditions and reproductive isolation across different regions have created spatially varying selective regimes that may be driving contemporary genetic differentiation. Shorter generation times in Southern clade blacklegged ticks may drive differences in their underlying evolutionary rates, likely contributing to higher effective population sizes and genetic diversity among Southern clade ticks, implying these ticks exhibit strong evolutionary potential (De et al. 2023; Frederick et al. 2023; Price 1980; Gandon and Michalakis 2002). Investigating how the evolutionary potential of different blacklegged tick populations influences local adaptation warrants further investigation.

The similar levels of genetic diversity observed in Minnesota and Pennsylvania tick populations indicate that All American clade ticks may have experienced divergent evolutionary histories. It is possible that (1) unknown migration routes or (2) undiscovered tick refugia during the glacial period harbored ancestral populations that seeded the Upper Midwest, contributing to the present‐day genetic structure. Previous research suggests that blacklegged ticks were confined to southeastern refugia following the last glacial maximum, later expanding northward along the Atlantic Coast as glaciers receded (Van Zee et al. 2015; Xu et al. 2020; Khatchikian et al. 2015). Northeastern populations are thought to have migrated westward, leading to the colonization of the Upper Midwest (Van Zee et al. 2015; Xu et al. 2020). Repeated expansion events would likely have reduced genetic diversity in Upper Midwestern populations compared to those in the Northeast and South. The patterns observed in this study and recent findings from Wisconsin (Gebremedhin et al. 2023) demonstrate that alternative demographic histories or ongoing gene flow must sustain genetic diversity across All American clade populations. Fossil evidence indicates that intact hardwood forests, present north of the Lower Mississippi River Basin during the last glacial maximum, could have served as a glacial refuge for displaced taxa (Soltis et al. 2006; McLachlan et al. 2005). Many animal species, including the primary reproductive host of blacklegged ticks, white‐tailed deer ( *Odocoileus virginianus* ), display phylogeographic structuring on either side of the Appalachian Mountains, with multiple southern refugia persisting during the last glacial period (Soltis et al. 2006). The eastern chipmunk ( *Tamias striatus* ), another typical blacklegged tick host (Cassens et al. 2023), was also shown to have survived near the Laurentide Ice Sheet throughout glaciation (Rowe et al. 2004). These factors indicate a more complex and nuanced demographic history of blacklegged ticks in the United States than previously appreciated. Validation across longitudinal and latitudinal gradients is needed, with transitional areas such as those in the Mississippi Valley and western Ohio Valley representing valuable targets for additional sampling (Foster et al. 2024). To elucidate whether gene flow or distinct evolutionary histories are the primary drivers of genetic patterns among All American clade ticks, further research should focus on these transitional regions to clarify the processes shaping their genetic diversity.

High‐impact variants unique to specific locations were present at low copy numbers, often resulting in frameshift mutations (Table 3 and Table S5). These rare (low copy number) high‐impact variants unique to tick populations may play roles in local adaptation, but it is equally plausible that some observed changes are neutral and not under selective pressure. Shared high‐impact variants between All American clade ticks from Minnesota and Pennsylvania were associated with genes involved in xenobiotic detoxification, nutrient metabolism, and signal regulation processes (Figure 2). It is important to note that these observations are based on gene ontology (GO) assignments and not a formal GO enrichment analysis, and adaptive interpretations should be treated with caution. Xenobiotic detoxification refers to recognizing, neutralizing, and eliminating harmful foreign substances ingested during blood meals (Palli 2020). Ticks have evolved various detoxification mechanisms to address these challenges, including enzymes and proteins such as cytochrome P450, glutathione S‐transferases, sulfotransferases, ATP‐binding cassette transporters, and esterases (De Araujo et al. 2024). Genes involved in xenobiotic detoxification and nutrient metabolism represent one of the most significantly expanded gene families in *Ixodes* ticks, reflecting their importance for obligate hematophagy (De Araujo et al. 2024). The prevalence of high‐impact variants in these genes could be partially attributed to the large number of gene copies within these families, rather than solely to adaptive pressures. Notwithstanding, the shared genetic variants in these genes among All American clade ticks may suggest differing selective pressures and evolutionary histories compared to Southern clade ticks. For instance, All American clade ticks inhabit more diverse environments and parasitize a broader range of host species than their Southern counterparts (Ginsberg et al. 2021; Ginsberg et al. 2017). Exposure to varied host chemical environments, heme concentrations, abiotic extremes, and acaricides may impose balancing selection on loci involved in xenobiotic detoxification. This selective pressure could maintain variants that enable blacklegged tick clades to neutralize a broader range of host‐derived and environmental toxins. The evolutionary history of gene families related to xenobiotic detoxification has been shown to differ between tick species inhabiting diverse ecological niches and parasitizing distinct host types (Jia et al. 2020; De Araujo et al. 2024; Wang et al. 2024; Le Gall et al. 2018). Disentangling adaptive signals from the influence of gene family expansions will require additional analyses, such as studying intraspecific evolutionary histories of these gene families and assessing their functional relevance.

Similarly, high‐impact variants in genes associated with transmembrane transport (Figure 2) may reflect both the adaptive and non‐adaptive consequences of expanded gene families. Transmembrane transport is crucial for the movement of nutrients, ions, water, and other molecules from the ingested blood meal across cell membranes during intracellular digestion (Whiten et al. 2018; Lara et al. 2015). Although these variants might enhance the capacity of All American clade ticks to manage diverse blood meal constituents from various host taxa, their presence could also result from the large number of genes in this category. The broader host range of All American clade ticks, including mammals and birds, compared to Southern clade ticks, which primarily parasitize lizards, could impose differing selective pressures on transport mechanisms (Ginsberg et al. 2021). Maintaining molecular diversity in transport mechanisms may allow ticks to metabolize or filter diverse blood meal components and facilitate nutrient uptake from hosts with varying blood chemistry. Further investigation is needed to clarify whether these variants confer specific functional advantages or are a byproduct of gene family expansion. Additionally, high‐impact gene variants related to sulfation (Figure 2) shared among All American clade ticks presents a similar case. As obligate blood feeders, ticks are subjected to vertebrate hemostatic mechanisms, including blood coagulation, which can impede blood meal acquisition and digestion (Thompson et al. 2017). Sulfation enhances the potency of anticoagulant proteins in tick saliva, aiding prolonged feeding periods and efficient blood meal acquisition (Franck et al. 2020). These variants might reflect adaptive responses to host‐specific hemostatic defenses, although the expanded nature of sulfotransferase gene families in tick genomes complicates direct adaptive explanations. Differential selective pressures on these genes could enable ticks to modulate anticoagulant potency in response to host‐specific challenges, potentially influencing feeding efficiency and host immunomodulation. Yet, without additional functional studies, it remains challenging to distinguish adaptive variation from neutral processes in these gene families. However, the observed genetic variation in xenobiotic detoxification, transmembrane transport, and sulfation genes underscores the complexity of tick genetic diversity. Genetic variation among blacklegged tick clades may provide evolutionary substrate for selection to act upon regarding host preference, colonization success, and the exploitation of diverse ecological niches, which may influence regional tick abundance patterns and shape disease transmission dynamics across the United States. Future studies incorporating GO enrichment analysis, functional assays, and comparative genomic approaches will be essential to elucidate the evolutionary and ecological significance of these variants.

While our study provides valuable insights into the genetic variations between the two clades of blacklegged ticks, several limitations should be considered when interpreting the results. Although the analysis identifies high‐impact variants, the phenotypic effects of these variants still need to be characterized. Investigating gene function through functional genomics will enhance our understanding of how specific genetic variants influence tick behavior and pathogen transmission. Furthermore, this study primarily focuses on tick populations from three locations, which may only capture part of the full extent of genetic variation across the entire geographic range of blacklegged ticks in the United States. The reliance on a single genome from each location (*n* = 1) constrains our ability to determine the full scope of genetic variation, potentially overlooking unique alleles or structural variants across diverse tick populations. We cannot be sure that the observed associations capture the full scope of genetic variation from these populations when considering individual ticks. To this end, differences in effective population sizes between the sampled populations may lead to inferences about molecular evolution, while the prevailing force is genetic drift. Expanding sampling efforts will facilitate a more comprehensive understanding of genetic diversity and its functional implications for tick‐borne disease transmission. Further, the available genetic resources limited our exploration of the genetic variation in *I. pacificus* , preventing whole genome variant calling for this species and subsequent comparison with *I. scapularis* . We chose to retain the *I. pacificus* mitochondrial and endosymbiont genomes in our analyses as it represents the closest North American relative to *I. scapularis* , providing context for phylogenetic comparisons. To minimize the potential for sequencing errors in our *I. scapularis* variant analysis, we utilized the highest accuracy base‐calling model that nanopore sequencing offers, imposed a strict minor allele frequency threshold, and excluded variants with poor mapping quality to enhance confidence in our variant calls. The strict filtering may exclude rare but true biological variants yet was necessary for handling nanopore sequencing data. Although our sequencing coverage for individual ticks ranged from 9× to 13×, studies have demonstrated that low‐coverage genome re‐sequencing can provide reliable data for genomic comparisons when coupled with strong statistical methods (Sims et al. 2014; Benjelloun et al. 2019). Despite these challenges, our study provides robust genetic and mitogenomic data for the tick research community to leverage in exploring *Ixodes* biology.

This study proposes that genetic variations play a crucial role in the evolution and adaptation of blacklegged ticks, influencing their vectorial capacity and contribution to tick‐borne diseases. By examining distinct genomic differences across All American and Southern clades, this work provides a foundation for understanding how genetic diversity may underlie phenotypic traits such as questing behavior, host preference, and pathogen transmission potential. These variations, especially in genes related to xenobiotic detoxification, transmembrane transport, and anticoagulant processes, highlight distinct adaptive strategies between clades, reflecting the selective pressures of their respective environments. Our findings underscore that the molecular adaptations observed could inform public health strategies to reduce tick‐borne disease risks. Future research can build on this study's genetic and mitogenomic data to more precisely determine how specific genetic variants influence vectorial traits, potentially guiding the development of targeted interventions and diagnostics for tick‐borne diseases.

## Author Contributions

**Jacob Cassens:** conceptualization (lead), data curation (lead), formal analysis (lead), funding acquisition (lead), methodology (lead), writing – original draft (lead), writing – review and editing (equal). **Adela S. Oliva Chávez:** writing – review and editing (equal). **Danielle M. Tufts:** writing – review and editing (equal). **Jianmin Zhong:** writing – review and editing (equal). **Christopher Faulk:** conceptualization (supporting), data curation (supporting), formal analysis (supporting), supervision (equal), writing – original draft (supporting), writing – review and editing (equal). **Jonathan D. Oliver:** conceptualization (supporting), supervision (equal), writing – original draft (supporting), writing – review and editing (equal).

## Conflicts of Interest

The authors declare no conflicts of interest.

## Supporting information


Data S1.

## Data Availability

Raw read fastq files, filtered VCF files, mitogenome FASTA files, endosymbiont FASTA files, bioinformatic pipelines, and Supporting Information have been submitted to Data Dryad (DOI: 10.5061/dryad.sbcc2frh8). Mitogenome assemblies have been submitted to GenBank (accession numbers: PQ557589‐PQ557592). Metadata are also stored in Data Dryad (DOI: 10.5061/dryad.sbcc2frh8). Bioinformatic pipelines and Supporting Information can also be found in the Supporting Information accompanying this manuscript.
