# Supplementary material for: Whole Genome Sequencing Reveals Clade‐Specific Genetic Variation in Blacklegged Ticks
Source: Ecol Evol. 2025 Feb 11;15(2):e70987. doi: 10.1002/ece3.70987 (PMC11814477; doi:10.1002/ece3.70987)
Supplement: Supplementary file 1 — Data S1. [file ECE3-15-e70987-s001.zip › bioinformatic_pipeline.html]

Whole genome sequencing reveals clade-specific genetic variation in blacklegged ticks


# Whole genome sequencing reveals clade-specific genetic variation in blacklegged ticks

#### Jacob Cassens

#### 2024-10-17

## Background

### Experiment

Authors: Jacob Cassens, Adela S. Oliva Chávez, Danielle M. Tufts,
Jianmin Zhong, Christopher Faulk, Jonathan D. Oliver

Samples: Ixodes scapularis ticks were collected from Minnesota (JC),
Pennsylvania (DMT), and Texas (ASOC); Ixodes pacificus ticks were
collected from California (JZ).

Extraction method: Qiagen MagAttract Kit (Cat. 67563)

Library prep: Native ligation sequencing kit (SQK-LSK-114)

## Analysis

### Concatenating raw read fastq files

As a pre-processing step, all fastq files generated from the
sequencing experiment were concatenated into a single fastq file for
each individual. Downstream analyses use the concatenated fastq
file.

```
cat *.fastq.gz > iscap_MN.fastq.gz
```

### Quality control on reads

Basic read statistics were generated using Nanoq.

```
nanoq -vvv -s -i iscap_MN.fastq.gz
```

### Variant calling

#### Mapping reads to reference genome

Raw reads were mapped to the PalLabHifi
reference genome using Minimap2. Mapped sam files
were converted to bam files, sorted, and indexed using samtools.

```
# Align to reference
minimap2 -ax map-ont pal_reference.fasta iscap_MN.fastq.gz -t 32 > iscap_MN_align_pal.sam
# Convert sam to bam file 
samtools view -S -b iscap_MN_align_pal.sam > iscap_MN_mapped_pal.bam
# Sort mapped bam file to removed unmapped reads
samtools view -b -F 4 iscap_MN_mapped_pal.bam > iscap_MN_aligned_pal.bam
# Index bam file
samtools index iscap_MN_aligned_pal.bam
```

#### Variant calling with Clair3

Variant calling was performed for individual ticks with the PalLabHifi
reference genome using Clair3. Clair3 requires an indexed
reference genome fasta file for processing.

```
# Index reference genome fasta file
samtools faidx pal_reference.fasta
# Run Clair3
./run_clair3.sh --bam_fn /home/jonathanoliverlab/jake/IS_MN/iscap_MN_aligned_pal.bam --ref_fn /home/jonathanoliverlab/jake/pal_reference.fasta --threads 24 --model_path /home/jonathanoliverlab/Clair3/models/ont --platform ont --output /home/jonathanoliverlab/jake/IS_MN --include_all_ctgs
```

### Variant statistics

#### Filtering variant call files

Variant call files (VCF) for individual ticks were filtered to retain
only those variants with frequencies >20% and quality scores >10%
using BCFtools.

```
bcftools view -i 'INFO/AF>=0.2 && QUAL>=30' iscap_MN.vcf -o iscap_MN_filtered.vcf
```

#### Creating pseudo alternative whole genome assemblies

VCFs were used to generate alternative whole genome assemblies using
the PalLabHifi
reference genome assembly using BCFtools.

```
bcftools consensus -f pal_reference.fasta -o alternative_genome_iscap_MN.fasta iscap_MN_filtered.vcf
```

#### Summary statistics on variant call files

Summary statistics for individual VCFs were generated using VariantQC.

```
java -jar DISCVRSeq.jar VariantQC \
     -R pal_reference.fasta \
     -V iscap_MN_filtered.vcf \
     -O iscap_MN_stats.html
```

#### Genome-wide heterozygosity and SNP density

Genome-wide heterozygosity and SNP density were calculated in 100 kb
windows using VCFtools.

```
# Heterozygosity
vcftools --vcf iscap_MN_filtered.vcf --window-pi 100000 --out iscap_MN_nucdiv_100kb
# SNP density
vcftools --vcf iscap_MN_filtered.vcf --SNPdensity 100000 --out iscap_MN_SNPden_100kb
```

#### Runs of homozygosity

Runs of homozygosity were calculated in 100 kb windows using Plink.

```
# Make bed files for PLINK
/Users/jtcassens/plink --vcf iscap_MN_filtered.vcf --make-bed --out iscap_MN_filtered_bed_out --allow-extra-chr

# Runs of homzygosity analysis
/Users/jtcassens/plink --bfile iscap_MN_filtered_bed_out --homozyg --homozyg-window-snp 100 --homozyg-window-het 1 --homozyg-gap 1000 --homozyg-kb 100 --homozyg-window-threshold 0.05 --homozyg-het 100 --homozyg-density 50 --homozyg-snp 25  --allow-extra-chr --out plink_analysis/iscap_MN/roh
```

#### Visualize SNP density, heterozygosity, and runs of homozygosity

SNP density, heterozygosity, and runs of homozygosity were visualized
by the fourteen longest scaffolds in the PalLabHifi
Ixodes scapularis reference genome using Circlize. Excel
spreadsheets containing data on each parameter are necessary input for
circlize.

```
mn <- read_excel("/Volumes/Xtreme/aim2/nuc_genome/test_circrlize.xlsx", sheet="iscap_MN")
mn_roh <- read_excel("/Volumes/Xtreme/aim2/nuc_genome/snpden_nucdiv_roh_stats.xlsx", sheet="iscap_MN_ROH")
## Initialize figure
circos.genomicInitialize(mn)

## Adding SNP density track
circos.trackPlotRegion(
  factors = mn$CHROM, 
  ylim = range(mn$`VARIANTS/KB`, na.rm = TRUE),  # Define y-axis limits
  panel.fun = function(x, y) {
    # Get the current sector index and its xlim
    sector_index <- get.cell.meta.data("sector.index")
    sector_xlim <- get.cell.meta.data("xlim")  # This gets the xlim of the current chromosome
    # Subset data for the current sector
    sector_data <- mn[mn$CHROM == sector_index, ]
    # Ensure the data is ordered by BIN_START
    sector_data <- sector_data[order(sector_data$BIN_START), ]
    # Plot the line with xlim set to the current chromosome's length
    circos.lines(sector_data$BIN_START, sector_data$`VARIANTS/KB`, type = "l", col='black', lwd = 0.25, area = TRUE)
  },
  track.height = 0.2
)
## Adding nucleotide diversity track
circos.trackPlotRegion(
  ylim = range(mn$PI, na.rm = TRUE),  # Define y-axis limits for PI
  panel.fun = function(x, y) {
    # Get the current sector index and its xlim
    sector_index <- get.cell.meta.data("sector.index")
    sector_xlim <- get.cell.meta.data("xlim")  # This gets the xlim of the current chromosome
    # Subset data for the current sector
    sector_data <- mn[mn$CHROM == sector_index, ]
    # Ensure the data is ordered by BIN_START
    sector_data <- sector_data[order(sector_data$BIN_START), ]
    # Plot the line with xlim set to the current chromosome's length
    circos.lines(sector_data$BIN_START, sector_data$PI, type = "l", col="navyblue", lwd = 0.25)
  },
  track.height = 0.2  # Adjust track height as needed
)
## Adding runs of homozygosity track
circos.trackPlotRegion(
  factors = mn_roh$CHROM,
  ylim = range(mn_roh$KB, na.rm = TRUE),  # Define y-axis limits
  panel.fun = function(x, y) {
    sector_index <- get.cell.meta.data("sector.index")
    sector_xlim <- get.cell.meta.data("xlim")
    # Subset data for the current sector
    sector_data <- mn_roh[mn_roh$CHROM == sector_index, ]
    sector_data <- sector_data[order(sector_data$BIN_START), ]
    # Plot the line with custom color and width
    circos.lines(sector_data$BIN_START, sector_data$KB, type = "h", col = "darkorange", lwd = 0.75)
  },
  track.height = 0.1
)
```

### Variant annotation

#### Annotating variants

Filtered VCFs were annotated using SnpEff. The current PalLabHifi
Ixodes scapularis reference genome is not natively supported by snpEff,
so a custom database was built for compatability. Extensive instructions
for creating the databases is detailed in the snpEff
documentation.

```
# Edit the snpEff config file
nano snpEff.config
# Insert the following text in the snpEff config file - do not use quotation marks around the hashtag below
"#" Ixodes scapularis, version PalLab 
Iscap.genome : iscap
# Create custom database
cd /path/to/snpEff
java -jar snpEff.jar build -gtf22 -v iscap
# Annotate with snpEff
java -jar snpEff.jar iscap_genome iscap_MN_filtered.vcf > iscap_MN.ann.vcf
```

#### Partition variants

Annotated variants were separated by the fourteen longest scaffolds
in the PalLabHifi
Ixodes scapularis genome and according to their impact/effect using snpSift.

```
# Filtering annotated variants by their effect
java -jar SnpSift.jar extractFields iscap_MN.ann.vcf CHROM POS REF ALT "ANN[*].EFFECT"
# Filtering annotated variants by their impact
java -jar SnpSift.jar extractFields iscap_MN.ann.vcf CHROM POS REF ALT "ANN[*].IMPACT"
```

### Gene ontology

#### Shared runs of homozygosity

To identify genes falling with shared runs of homozygosity, we
intersected shared runs of homozygosity with the position of annotated
genes from the PalLabHifi
reference genome annotation file (GTF) using dplyr in R.

```
# Load packages
library(dplyr)
library(readxl)
# Read in excel data
vars <- read_excel("/Volumes/Xtreme/aim2/nuc_genome/excel/high_impact_variants.xlsx", sheet="PalLabHifi_gene_annotation_IDs")
roh <- read_excel("/Volumes/Xtreme/aim2/nuc_genome/excel/roh_examination.xlsx", sheet="roh_data")
# Run analysis by chromosome
vars_chr1 <- vars %>% filter(CHROM == "NW_024609835")
roh_chr1 <- roh %>% filter(CHROM == "NW_024609835")
vars_chr1$In_Range <- FALSE
for (i in 1:nrow(vars_chr1)) {
  # Check if the POS value falls within any BIN_START and BIN_END in roh_chr1
  vars_chr1$In_Range[i] <- any(vars_chr1$BIN_START[i] >= roh_chr1$BIN_START & vars_chr1$BIN_END[i] <= roh_chr1$BIN_END)
}
vars_chr1_in_range <- vars_chr1 %>%
  filter(In_Range == TRUE)
View(vars_chr1_in_range)
```

The resulting gene IDs were placed into an excel spreadsheet with
their chromosomal identity, and start and end positions. Gene IDs were
then copied (as a list) and pasted into DAVID’s gene ontology prediction
tool.

#### Visualization

Gene ontology terms shared among individuals within shared runs of
homozygosity identified through DAVID were visualized using ggplot2 in R.

```
library(ggplot2)
library(readxl)
ibd_go <- read_excel("/Volumes/Xtreme/aim2/nuc_genome/excel/ibd_analysis.xlsx", sheet="shared_roh_gene_ID")
ibd_plot <- ggplot(ibd_go, aes(x = reorder(Term, Count), y = Count, fill = Category)) +
  geom_bar(stat = "identity") +
  coord_flip() + # Flip coordinates to make it vertical
  scale_fill_manual(values = c("Biological process" = "#117733", 
                               "Cellular component" = "#BB2818", 
                               "Molecular function" = "#0072B2")) +
  labs(x = "", y = "# of genes", 
       title = "",
       fill = "") +
  theme_minimal() +
  theme(axis.text.y = element_text(size = 10, color = "black"),
        axis.text.x = element_text(size = 10, color = "black"),
        axis.title.x = element_text(size = 12, , face = "bold", color = "black"),
        plot.title = element_text(size = 14, color = "black"),
        panel.grid.major = element_blank(),
        panel.grid.minor = element_blank(),
        panel.border = element_blank(),
        axis.line.x = element_line(color = "black"),
        axis.line.y = element_line(color = "black"),
        axis.ticks.x = element_line(color = "black"),
        axis.ticks.length = unit(0.3, "cm"), 
        #legend.position = c(0.9, 0.1),
        legend.text = element_text(size = 12, color = "black"),
        plot.background = element_rect(fill = "white")) +
  scale_y_continuous(breaks = seq(0, max(ibd_go$Count), by = 10), expand = expansion(mult = c(0, 0.05)))
```

#### Comparing high impact variants

Evaluation of genes with high impact variants unique to individuals,
shared among individuals, or shared between all individuals was
determined by intersecting gene IDs from all three individuals, copying
these gene IDs into DAVID’s
gene ontology prediction tool, and reported as GO terms using dplyr in R.

```
# Load packages
library(dplyr)
library(readxl)
# Read in data
A <- read_excel("/Volumes/Xtreme/aim2/nuc_genome/excel/MN_test_GO_analysis.xlsx", sheet="iscapMN_highimpact_gene_IDs")
B <- read_excel("/Volumes/Xtreme/aim2/nuc_genome/excel/MN_test_GO_analysis.xlsx", sheet="iscapPA_highimpact_gene_IDs")
C <- read_excel("/Volumes/Xtreme/aim2/nuc_genome/excel/MN_test_GO_analysis.xlsx", sheet="iscapTX_highimpact_gene_IDs")
# Specify the terms to intersect as gene ID
mn_terms <- A$ID
pa_terms <- B$ID
tx_terms <- C$ID
# Identifying gene IDs shared among all individuals
shared_all <- intersect(intersect(mn_terms, pa_terms), tx_terms)
shared_all
# Identifying gene IDs unique to Minnesota
unique_to_mn <- setdiff(mn_terms, union(pa_terms, tx_terms))
unique_to_mn
# Identifying gene IDs unique to Pennsylvania
unique_to_pa <- setdiff(pa_terms, union(mn_terms, tx_terms))
unique_to_pa
# Identifying gene IDs unique to Texas
unique_to_tx <- setdiff(tx_terms, union(mn_terms, pa_terms))
unique_to_tx
# Identifying gene IDs shared between Minnesota and Pennsylvania
shared_mn_pa <- intersect(mn_terms, pa_terms)
shared_mn_pa
# Identifying gene IDs shared between Minnesota and Texas
shared_mn_tx <- intersect(mn_terms, tx_terms)
shared_mn_tx
# Identifying gene IDs shared between Pennsylvania and Texas
shared_pa_tx <- intersect(pa_terms, tx_terms)
shared_pa_tx
```

#### Visualization of comparison

Gene ontology terms unique to individuals, shared among individuals,
or shared between all individuals, were extracted from the list above,
placed into a new excel spreadsheet, and visualized using ggVennDiagram and
ggplot2 in R.

```
# Load packages
library(ggVennDiagram)
library(ggplot2)
# Create GO terms list for ggVennDiagram - it requires a special format
GO_terms_list <- list(
  Minnesota = A$ID,
  Pennsylvania = B$ID,
  Texas = C$ID
)
# Create Venn diagram comparing individuals
ggVennDiagram(GO_terms_list, label_alpha = 0) + 
  scale_fill_gradientn(colors =  c("#8da0cb", "#1b9e77","#b1686e","#f2f0c4","#669564","#bfb7be","#c07328")) + 
  theme(legend.position = "none",
        text = element_text(size = 20))
# Barplot for biological process GO terms, no. of genes >5
go_comp_bp <- read_excel("/Volumes/Xtreme/aim2/nuc_genome/excel/MN_test_go_analysis.xlsx", sheet="go_comp_>5_bp")
go_bar_bp <- ggplot(go_comp_bp, aes(x = count, y = reorder(term, count), fill = status, pattern = category)) +
  geom_bar(stat = "identity", show.legend = FALSE) + 
  labs(x = "Number of Genes", y = "GO Term", title = "Biological process") +
  theme_minimal() +
  theme(
    axis.text.y = element_text(size = 10, color = "black"),
    axis.title.y = element_blank(),
    axis.text.x = element_text(size = 10, color = "black"),
    axis.title.x = element_text(size = 12, face = "bold", color = "black"),
    plot.title = element_text(size = 14, color = "black", face = "bold"),
    panel.grid.major = element_blank(),
    panel.grid.minor = element_blank(),
    panel.border = element_blank(),
    axis.line.x = element_line(color = "black"),
    axis.line.y = element_line(color = "black"),
    axis.ticks.x = element_line(color = "black"),
    axis.ticks.length = unit(0.3, "cm"),
    legend.text = element_text(size = 10, color = "black"),
    legend.title = element_blank(),
    legend.position = "bottom",
    strip.text = element_text(size = 12, color = "black", face = "bold")  # Style for facet labels
  ) +
  scale_fill_manual(
    values = c("MN_PA_TX" = "#669564", "MN_TX" = "#c07328", "MN_PA" = "#b1686e", "PA_TX" = "#1b9e77", "MN" = "#f2f0c4", "PA" = "#bfb7be", "TX" = "#8da0cb"),
    labels = c("MN_PA_TX" = "MN, PA, TX", "MN_TX" = "MN, TX", "MN_PA" = "MN, PA", 
               "PA_TX" = "PA, TX", "MN" = "MN", "PA" = "PA", "TX" = "TX"), # Custom legend labels
    guide = "none"
  ) +
  scale_x_continuous(expand = c(0, 0))
# Barplot for molecular function GO terms, no. of genes >5
go_comp_mf <- read_excel("/Volumes/Xtreme/aim2/nuc_genome/excel/MN_test_go_analysis.xlsx", sheet="go_comp_>5_mf")
go_bar_mf <- ggplot(go_comp_mf, aes(x = count, y = reorder(term, count), fill = status, pattern = category)) +
  geom_bar(stat = "identity", show.legend = FALSE) + 
  labs(x = "Number of Genes", y = "GO Term", title = "Molecular function") +
  theme_minimal() +
  theme(
    axis.text.y = element_text(size = 10, color = "black"),
    axis.title.y = element_blank(),
    axis.text.x = element_text(size = 10, color = "black"),
    axis.title.x = element_text(size = 12, face = "bold", color = "black"),
    plot.title = element_text(size = 14, color = "black", face = "bold"),
    panel.grid.major = element_blank(),
    panel.grid.minor = element_blank(),
    panel.border = element_blank(),
    axis.line.x = element_line(color = "black"),
    axis.line.y = element_line(color = "black"),
    axis.ticks.x = element_line(color = "black"),
    axis.ticks.length = unit(0.3, "cm"),
    legend.text = element_text(size = 10, color = "black"),
    legend.title = element_blank(),
    legend.position = "bottom",
    strip.text = element_text(size = 12, color = "black", face = "bold")  # Style for facet labels
  ) +
  scale_fill_manual(
    values = c("MN_PA_TX" = "#669564", "MN_TX" = "#c07328", "MN_PA" = "#b1686e", "PA_TX" = "#1b9e77", "MN" = "#f2f0c4", "PA" = "#bfb7be", "TX" = "#8da0cb"),
    labels = c("MN_PA_TX" = "MN, PA, TX", "MN_TX" = "MN, TX", "MN_PA" = "MN, PA", 
               "PA_TX" = "PA, TX", "MN" = "MN", "PA" = "PA", "TX" = "TX"), # Custom legend labels
    guide = "none"
  ) +
  scale_x_continuous(expand = c(0, 0))
```

### Mitogenome

#### Assembly, polishing, and annotation

Mitogenome assembly and annotation was performed using MitoHiFi on Galaxy. Briefly, the user will upload
their raw sequence data into Galaxy, and navigate to the MitoHiFi
tool. The user will select ‘Run MitoHiFi’, choose their input mode,
upload the reference mitogenome in fasta and genbank format, select
‘Invertebrate genetic code’, and select ‘Generate an output package in
ZIP format. The tool will then produce output in a zipped file, which
can be extracted. The draft assembly contained in the zipped file was
polished twice using Medaka. After
polishing the mitogenome assembly, it was annotated again using Mitos2
on Galaxy to ensure concordance
with the initial annotation. Briefly, the user will upload their
polished mitogenome into Galaxy and
navigate to the Mitos2 tool. The user will upload their polished
mitogenome assembly, select the ’Invertebrate genetic code’, select the
‘RefSeq89 Metazoa’ reference data, and select the ‘BED’ and ‘nucleotide
FASTA’ outputs. All annotations were then verified using Geneious. The annotated mitogenome
was then uploaded to OGDRAW for
constructing organelle genome maps.

As an alternative to this method, you can align your raw reads to a
reference mitogenome using Minimap2, parse and sort the
mapped reads using samtools,
assemble the mapped reads using Flye, polish the draft
assembly using Medaka, and annotate
the draft assembly with Mitos2. Both methods were used in this analysis,
and the latter is described here.

```
# Map reads to reference mitogenome
minimap2 -ax map-ont mitogenome_reference.fasta iscap_MN.fastq.gz -t 32 > iscap_MN_align_mito.sam
# Parse and sort mapped reads
samtools view -S -b iscap_MN_align_mito.sam | samtools view -b -F 4 - | bam2fastq -o iscap_MN_aligned_mito.fastq
# Assemble mitogenome using flye
flye --nano-hq iscap_MN_aligned_mito.fastq --out-dir flyeout --genome-size 0.016m --threads 128 --asm-coverage 50
# Polish mitogenome assembly
medaka_consensus -i iscap_MN.fastq.gz -d flyeout/assembly.fasta -o . -t 2
```

The final polished assembly can then be uploaded into Galaxy and
input into Mitos2 for annotation, specifying for metazoan RefSeq and
invertebrate genetic code.

#### Phylogenetics

Phylogenetic analysis was performed using Geneious. Briefly, our final
mitogenome assembly for each individual was imported into Geneious. Mitogenome assemblies for
all reference Ixodes and Amblyomma species were downloaded from NCBI and
imported into Geneious.
Mitogenome sequences were then aligned by clicking the
‘Pairwise/Multiple Align..’ tool and selecting Clustal Omega as the
aligner. The resulting alignment was then used as input for the
phylogenetic tree by selecting ‘Tree’, clicking ‘RAxML’, and choosing
the following parameters: ‘GTR GAMMA I’ model, ‘Rapid Bootstrapping and
search for best-scoring ML tree’ algorithm, ‘1,000’ number of boostrap
replicates, and ‘12345’ parsimony random seed.

### Endosymbiont

#### Assembly

Endosymbiont assembly was performed by mapping raw reads to the PalLabHifi
Rickettsia buchneri genome using Minimap2. Mapped reads were
parsed and sorted using samtools.
Mapped reads were assembled using Flye, specifying tags
for high quality reads, genome size of 1.7Mb, the number of cores to
utilize, and the lower threshold on coverage per base (50x). The draft
assembly was then polished using Medaka, and the final
assembly was used for phylogeny analysis.

```
# Map reads to reference mitogenome
minimap2 -ax map-ont rickettsia_buchneri_reference.fasta iscap_MN.fastq.gz -t 32 > iscap_MN_align_endosymbiont.sam
# Parse and sort mapped reads
samtools view -S -b iscap_MN_align_endosymbiont.sam | samtools view -b -F 4 - | bam2fastq -o iscap_MN_aligned_endosymbiont.fastq
# Assemble mitogenome using flye
flye --nano-hq iscap_MN_aligned_endosymbiont.fastq --out-dir flye_out --genome-size 1.7m --threads 128 --asm-coverage 50
# Polish mitogenome assembly
medaka_consensus -i iscap_MN.fastq.gz -d flyeout/assembly.fasta -o . -t 2
```

#### Phylogenetics

Phylogenetic analysis of the commensal bacteria found in each
individual tick followed the protocol outlined above for the
mitogenomes.
